# Dose consumption for quality assurance and maintenance with a dedicated IORT accelerator

**DOI:** 10.1120/jacmp.v10i4.2292

**Published:** 2009-10-27

**Authors:** Frank W. Hensley

**Affiliations:** ^1^ Department of Radiation Oncology University Clinics Heidelberg 69120 Heidelberg Germany

**Keywords:** intraoperative radiotherapy, dedicated IORT accelerators, quality assurance, radiation protection

## Abstract

Dedicated accelerators for intra‐operative radiation therapy (IORT) are operated at high dose rates in order to achieve short treatment times within which the anaesthetisized patient must be remotely monitored (e.g. via video cameras and telemetric anesthesia instruments). Due to these high dose rates, large doses accumulate from the irradiations necessary for quality assurance (QA) and maintenance. In practice, the dose load for QA, maintenance and repairs will probably far exceed the patient dose. The total dose consumption for all of these actions must be considered in facility licensing, in radiation protection assessments, and in the shielding calculations. Dose consumption for QA and maintenance was assessed for the dedicated IORT facility at Heidelberg University for the operation period between June 1991 and December 2007 (15.5 years). Average doses per year of 5847 Gy for maintenance and repairs and 3686 Gy for QA were needed during this period. The causes and composition of these high doses are analyzed and discussed separately for irradiations that need to be performed in the operation room and which, with a mobile accelerator, may be performed in a separate QA vault.

PACS number: 87.56.bd

## I. INTRODUCTION

Dedicated accelerators for intra‐operative radiation therapy (IORT) are operated at high dose rates in order to achieve short treatment times within which the anesthetized patient can only be remotely monitored via video cameras and telemetric anesthesia instrumentation. Due to these high dose rates, large doses accumulate from the irradiations necessary for quality assurance (QA) and maintenance. In practice, the dose load for QA, maintenance, and repairs will probably far exceed the patient dose. The total dose consumption for all of these actions must be considered in facility licensing, in radiation protection assessments, and in the shielding calculations. For a stationary linac, the shielding of the operation room (OR) must be dimensioned to reduce the dose to the surrounding area to values commensurate with maximal levels permitted by national and international regulations^(^ ^1^ ^,^ ^2^ ^)^ applying the ALARA principle.^(3)^ In many cases (e.g. if the adjacent area is in the public domain or strongly frequented by uncontrolled persons such as varying nursing and anesthesia staff, guests, students, possibly pregnant persons), it may be prudent to limit dose to the maximal public dose of 1 mSv per year. Commissioning of a new accelerator causes an additional dose load which must be provided for in the respective year. For the newly designed mobile IORT accelerators,^(^ ^4^ ^,^ ^5^ ^,^ ^6^ ^,^ ^7^ ^)^ those parts of the dose that can be scheduled in advance can be performed in a shielded vault separate from the OR. This vault must also be licensed, and its shielding must be assessed in the planning phase. Few data are available in literature to estimate total dose loads and radiation protection in IORT.^(^ ^8^ ^,^ ^9^ ^,^ ^10^ ^)^ Commissioning and periodic quality assurance of medical linear accelerators are required by the licensing and regulatory officials. Recommendations for the appropriate measurements and allowable machine tolerances are given in international and national standards and in guidelines formulated by professional societies.^(^ ^11^ ^–^ ^17^ ^)^ Special recommendations for IORT were published by AAPM Task Groups (TG) 48 and 72,^(^ ^14^ ^,^ ^15^ ^)^ and by the Italian National Health Institute.^(17)^ The measurements analyzed in this work were performed applying the of the German DIN standards,^(16)^ which in most items are identical to those recommended by AAPM TG‐40.^(12)^ Some additional IORT specific items (e.g. cone factors, gap factors) were commissioned according to the recommendations by AAPM TG‐48.^(14)^ Dose consumption for QA and maintenance was assessed for the dedicated IORT facility at Heidelberg University for the operation period between June 1991 and December 2007 (15.5 years). Average doses per year of 5847 Gy for maintenance and repairs and 3686 Gy for QA were needed in this period. The causes and composition of these high doses are analyzed and discussed.

## II. MATERIALS AND METHODS

Dose consumption for the dedicated IORT accelerator Mevatron ME at Heidelberg University was calculated and compared for the major radiation causes. The causes considered are:

- Patient treatment
- Commissioning
- Quality assurance and dosimetry
- Maintenance and repairs, and
- Legally required periodic safety inspections

All doses given in this text are specified at the maximum of the depth dose curve in water at the normal focus‐to‐surface distance of 100 cm. The accelerator's dose monitor calibration sets 10 monitor units (MUs) equal to a dose of 1 Gy for the reference (12 cm diameter round) applicator.

The dose for patient treatment was taken from the treatment records or, where not known, calculated by multiplying the number of patients treated in each year by an average dose of 12.5 Gy, the dose typically given at Heidelberg. Doses for repairs and maintenance were calculated using the reading of the high voltage (HV) timer. On the Mevatron ME, the HV timer records the time when voltage is applied to the electron injection system and, therefore, correlates to beam on time. The difference between HV time at the end and the beginning of the measurements as noted in the system log book was multiplied by an average dose rate. The Mevatron ME operates at three dose rates of 3 Gy/min, 6 Gy/min, and 9 Gy/min. Since most of the works during commissioning, periodic quality checks, and maintenance must be performed for all three dose rates, an average rate of 6 Gy/min was used for the calculations in all cases where the correct dose rate is not known. Many beam adjustments are performed at the highest dose rate, where the machine is routinely operated in patient treatments and where the settings are most critical, and only controlled at the lower dose rates. For these actions, a dose rate of 9 Gy/min was used in the calculations.

For the commissioning works, the records allow a nearly exact summation of the doses used for all performed measurements. This number can be compared to the dose calculated from the HV times for the same set of measurements, and can thus be used to check and confirm the validity of the calculations using the HV timer.

No complete log of the HV timer was kept for QA procedures. Therefore, the doses for these items were calculated by multiplying the number of QA procedures with the average dose required for the respective action. For daily QA, the number of recorded checks was used in this calculation; for the other procedures, dose is calculated using the number of procedures required by the German DIN standards.^(16)^

**Figure 1:**
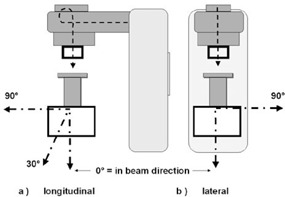
Set‐up for the leakage measurements: (a) measuring positions in the plane of the bending magnet = longitudinal to the acceleration direction; (b) measuring positions 90° lateral to the plane of the bending magnet.

To relate the assessed doses to ambient doses around the machine and to give a base for shielding calculations, leakage radiation around the Mevatron ME was measured in 1 m distance from the intersection of the beam axis with a 30 cm by 30 cm by 15 cm polystyrene phantom at four positions (shown in Fig. 1). Measurements were performed for all energies with the largest (12 diameter) applicator using a 1000 cm^3^ ionization chamber (PTW M32002, PTW Freiburg, Lörracher Str. 7, 79115 Freiburg, Germany).

In beam forward direction, an additional measurement was performed with a 10 cm phantom thickness to simulate the effect of reduced absorption for irradiations with less tissue in the beam.

### A. The Mevatron ME

The Mevatron ME (Siemens Medical Systems, 4040 Nelson Avenue, Concord CA 94520, USA) is a dedicated electron‐only accelerator designed especially for IORT.^(^ ^10^ ^,^ ^18^ ^)^ Its design is based on the Siemens Mevatron M series, a radiotherapy accelerator with analog electronic controlling using a Magnetron as RF generator. The Mevatron ME is adapted to IORT needs by removing all components for X‐ray production. In the treatment head, several heavy parts are omitted in order to reduce bremsstrahlung production (e.g. the moving collimator jaws). Furthermore, all motor drives in the head are omitted or placed in a separate machine room to reduce the explosion hazard due to anesthesia gases in the OR.

The accelerator produces electron beams with six energies (6, 8, 10, 12, 15, 18 MeV), each at three dose rates 3, 6, 9 Gy/min.

Typical treatment dose rate is 9 Gy/min in order to keep the time short when the patient can only be remotely controlled by the anesthetist. The other two dose rates are scarcely used for treatment (e.g. to irradiate small doses) but are kept operational as backup.

Energy analysis and stabilization of the Mevatron ME is performed with a 270° bending magnet; beam fattening is achieved by dual scattering foils.

The Mevatron ME has a simplified collimator system consisting of only a conical primary collimator. Additional collimator presetting for each applicator size is achieved by circular double plane diaphragm rings called annuli, which are inserted into the accessory holder.^(^ ^14^ ^,^ ^19^ ^,^ ^20^ ^)^ Final beam collimation is provided by (typically cylindrical) applicators, which are fixed to the patient table with a bookwalter clamp assembly and have no mechanical connection to the accelerator (soft docking). This system effectively excludes forces during the docking process which could lead to patient injury.

### B. Soft (air) docking

**Figure 2:**
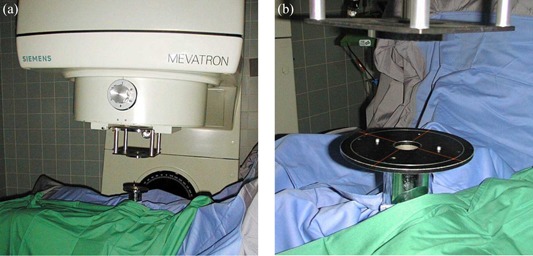
(a) shows the Mevatron ME in operation; (b) shows the laser pointers used for soft docking.

Beam alignment with the applicator is achieved by a set of eight laser pointers mounted in the head of the accelerator. The intersection of four point lasers with four line lasers on a ring on the entrance plane of the applicator indicates correct beam alignment and focal distance.^(^ ^14^ ^,^ ^19^ ^)^ (see Figure 2(a) and 2(b))

## III. RESULTS

The following paragraphs describe the doses evaluated for the considered QA and maintenance actions. Some of the items are performed only once (e.g. commissioning, development of applicators) and contribute only to limited plannable periods, for which possibly special arrangements like restrictions of access to the OR and surrounding rooms can be organized. Other actions must be repeated periodically, and provision must be made to limit the dose to the public. The doses for different actions are displayed in Tables 1–4 in groups, which can be separately added for those irradiations which must be performed in the OR and those which can possibly be performed in a separate vault with a mobile accelerator.

### A. Commissioning

A total dose of 5145 Gy for commissioning was estimated using the following assumptions for the named measurements. The doses for commissioning are summarized in Table 1. As discussed below, the dose consumed for commissioning is equivalent to the dose of 412 patients with an average treatment dose of 12.5 Gy or four years of operation at 100 patients per year.

**Table 1:** Doses consumed for commissioning of a dedicated IORT accelerator.

	*No. Items*	*Dose per Item*	*Total Dose*
PDDs	16 applicators 6 energies	1.5 Gy / curve	144 Gy
OARs	16 applicators 6 energies 6 depths in/crossplane	3 Gy / curve	3456 Gy
**Monitor Calibration**	6 energies 3 dose rates 3 repeats	5 Gy / measurement	270 Gy
**Cone Factors**	16 applicators 6 energies	15 Gy / applicator	240 Gy
**Gap Factors**	3 applicators 5 distances 6 energies	15 Gy / applicator and energy	270 Gy
**Monitor Linearity**	4 doses 3 dose rates 2 energies	87 Gy / energy	522 Gy
**Monitor Stability**	1 dose 6 energies 4 measurements	3 Gy / measurement	72 Gy
**Beam Stability at all Gantry Angles**	PDD in/crosspl. OAR 3 applicators 6 energies	1.5 / 3 Gy per curve	135 Gy
**Open Profiles**	2 profiles 6 energies	3 Gy / profile	36 Gy
***Total***			5145 Gy

#### A.1 PDDs and OARs

To provide an isodose atlas for all possible situations during IORT, commissioning included comprehensive dosimetry for:

- every available energy
- every available applicator
- and for a number of situations deviating from normal setup (gaps between applicator end and patient, deviating focus‐to‐target distances, field shapes altered by shielding)

At time of commissioning, the Heidelberg Mevatron ME provided:

- 6 electron energies (6, 8, 10, 12, 15, 18 MeV)
- 16 different applicators: 8 straight round applicators (diameters 5, 6, 7, 8, 9, 10, 11, 12 cm), 8 beveled round applicators (same diameters, one bevel angle of 22.5°)

It was therefore necessary to measure:

- 6×16=96 central axis depth dose (PDD) curves, and
- 6×16×2 (in‐/crossplane) distributions of off‐axis‐ratio (OAR) curves

Each OAR curve was measured at six depths (surface (0.5 cm), $D_{\max},\;0.9\;D_{\max},\;0.8\;D_{\max},\;0.5\;D_{\max},\;0.1\;D_{\max}$).

In total, 96 PDD plus 1152 OAR curves were measured for this item.

To reduce total dose, all the commissioning measurements were performed at a dose rate of 3 Gy/min. To achieve sufficient statistics, dose was integrated for 0.6 sec at each measuring point on the curves using a Markus chamber in a PTW MP3 3‐dimensional water phantom. Average drive time between two positions was estimated as 0.4 sec, giving 1 sec. of beam time for each measuring point.

- Each PDD curve was measured at 16–25 positions so that an average measuring time of 0.5 min/curve was assumed, thus consuming 1.5 Gy per curve. These assumptions yield a total dose consumption of 144 Gy for the measurement of the PDD curves.
- Each OAR curve was measured at 45–55 positions averaging to 1 min/curve or 3 Gy per curve. These assumptions yield a total dose consumption of 3456 Gy for the measurement of the OAR curves.

Note that dose rates 3, 6, 9 Gy/min are available. Since the measuring time per position should not be further reduced (e.g. in order to avoid artifacts from water motion), the doses per curve will grow proportionally with dose rate (e.g. will be higher by a factor of 3 if one decides to measure at the treatment dose rate 9 Gy/min).

#### A.2 Monitor calibrations

Monitor calibrations were performed for six energies and at all three dose rates. A dose of approximately 5 Gy was used for each measurement. Each calibration was repeated at least three times. The calibration measurements therefore consumed a total dose of 6×3×3×5Gy=270Gy.

#### A.3 Cone factors

To measure cone factors, a dose of approximately 15 Gy was used for each of 16 applicators (including 3 repeats). These measurements therefore consumed a total dose of 16×15Gy=240Gy.

#### A.4 Gap factors

To correct for gaps between the applicator end and the beam entrance into tissue, gap factors were measured for the smallest (5 cm), the largest (12 cm), and an intermediate size (8 cm) applicator at five distances each. Measurements were performed at all six energies. A dose of 15 Gy is assumed for each applicator, including repeats. These measurements therefore consumed a total dose of 3×6×15Gy=270Gy.

#### A.5 Monitor linearity

Monitor linearity was checked for two energies (highest and lowest), for doses of 2, 5, 10, 20, and 50 Gy, and at all dose rates. These measurements consumed a total dose of 2×3×87Gy=522Gy.

#### A.6 Monitor stability during the day

To confirm the constancy of the monitor calibration over long time periods, measurements of 3 Gy were made at four times during one day for all energies. These measurements consumed a total dose of 4×6×3Gy=72Gy.

#### A.7 Beam stability at different gantry angles

To confirm beam stability at all gantry angles (e.g. under the influence of the geomagnetic field), PDDs and inplane and crossplane OARs at depth of $D_{\max}$ were measured for three applicators (5 cm, 8 cm, 12 cm) at three different gantry angles (0°, 90°, 324° ^1^) for all energies. Using the same dose assumptions as otherwise for PDDs and profiles, these measurements consumed a total dose of 3×6×(1.5+2×3)Gy=135Gy.

#### A.8 Open profiles

Beam profiles (in/crossplane) without applicator were measured for all energies. These measurements consumed a total dose of 2×6×3Gy=36Gy.

### B. Comparison of calculation methods

The dose of 5145 Gy calculated for commissioning by the method above can be compared to a dose estimate of 4601 Gy calculated from the HV timer difference of 25.56 hours recorded before and after the commissioning measurements, assuming a dose rate of 3 Gy/ min at all times. This difference is probably due to an overestimation of the measuring times in the calculations above (mainly the drive‐times between measuring positions), but possibly also to some shortcuts and omissions in the measuring program^2^. To stay on the conservative side (some irradiations were also performed at higher dose rates), the larger dose (5145 Gy) is used in the following discussion.

### C. Development of new cones

At a later time, a set of six squircle (horseshoe‐shaped) cones^(21)^ (5×9,6×7,7×8,10×11,10×13,11×14cm=diameterofcircularend×totallength) and 12 additional round + beveled cones at 0.5 cm steps between the existing cones (diameters 6.5, 7.5, 8.5, 9.5, 10.5, 11.5 cm) were built and commissioned. Using the same assumptions as above, this development required the same dose consumption for PPDs, OARs and cone factors as during general commissioning, yielding a total of 4320 Gy. These doses are summarized in Table 2.

**Table 2:** Doses consumed for the commissioning of 18 new applicators.

	*No. Items*	*Dose per Item*	*Total Dose*
PDDs	18 applicators 6 energies	1.5 Gy/ curve	162 Gy
OARs	18 applicators 6 energies 6 depths in/crossplane	3 Gy / curve	3588 Gy
**Cone Factors**	18 applicators 6 energies	15 Gy / applicator	270 Gy
***Total***			4320 Gy

### D. Periodic dose consumption during regular operation

The doses needed for periodic quality controls are summarized in Table 3, those for repairs and maintenance in Table 4. The tables show that on average 3686 Gy per year were needed for quality assurance (assuming a safety plus calibration check of 8 Gy on every day a patient is scheduled), and 5847 Gy for repairs and maintenance.

**Table 3:** Doses for periodic quality assurance measurements at a dedicated IORT accelerator.

*Items checked*	*Dose per item*	*Total dose per check*	*Period*	*Dose per year (average)*	*Dose per week (average)*	*% Of average annual patient dose*
**DAILY PROCEDURES**							
**Daily safety checks**	Beam interrupt by:						
	– primary/secondary monitor	3 Gy					
	– beam timer	1 Gy	4 Gy	daily	538 Gy	10.4 Gy	61%
	– Rad Off button^a^	−					
	Beam can be re‐started after Rad Off						1check=32% average dose for 1 pat.
	Functionality of:						
	– door interlocks^a^						
	– safety interlocks^a^ (annulus	−					
	insertion correct annulus for	−					
	applicator correct fattening filter)	−					
	– warning signs^a^	−					
	– staus/ interlock indicators^a^	−					
	– dose calculator circuit^b^	−					
	– motion stop	−					
	– laser indicators for soft docking	−					
	– video patient monitoring	−					
	***Status of sterile accessories***	−					
	Technical parameter:						
	– machine temperature	−					
	– cooling water level	−					
	– cooling water temperature	−					
	$-\;{\text{SF}}_{6}$ pressure in RF guide (1 dose at 2 energies)	−					
**Daily monitor check**		2 Gy	4 Gy		538 Gy	10.4 Gy	61%
**Total dose daily procedures**			Σ : 8 Gy		Σ :1080 Gy	Σ:20.8	Gy Σ : 122%
**Biweekly monitor calibration**	1 measurement at 6 energies/3 dose rates	3 Gy / 6 Gy	72 Gy	**biweekly**	1800 Gy		203%
**Constancy of beam energy**	1 film exposure at 6 energies	0.8 Gy	4.8 Gy	**4 per yr**	19.2 Gy		2.2%
**ANNUAL PROCEDURES**							
**Annual**	PDD and in/crossplane						
**Calibration**	OAR at $D_{\max}$ for:						
	−2applicators	1.5 GY/PDD	270 Gy	1 per yr	270 Gy		31%
	−6energies	3 Gy/OAR					
	−3 gantry angles (0°, 90°, 270°)		216 Gy		216 Gy		
	**Monitor calibration in water + cross calibration of polystyrene phantom**	3Gy/measurement 1 repretition	Σ : 486 Gy		Σ : 486 Gy		24% Σ : 55%
	−1 standard applicator						
	−3 dose rates						
	−6 energies						
	−2 phantoms						
**Annual safety check**	Test of all safety and system interlocks		325 Gy (average)	1 per yr	325 Gy		37%
**Total dose, periodic QA**							
w.4 Gy daily mon. check					3706 Gy		418%
w.12 Gy daily mon. check					4774 Gy		539%

^a^ These items are checked during test of monitor functionality and, therefore, consume no additional dose.

^b^ The dose calculator of the Mevatron ME is an electronic circuit, which calculates the preset of the secondary dose monitor and the beam timer for the entered monitor units. A fault in this circuit frequently causes the electronics to calculate no secondary presets so that the beam is suppressed by interlock. This malfunction can be corrected by machine reset, and is tested before the functionality checks of the beam monitors.

**Table 4:** Doses for repairs, maintenance, and quality assurance with a dedicated IORT accelerator.^a^

	*Max*	*Min*	*Average*	*Average Ratio to Annual Patient Dose*
**Repairs**	6216 Gy	222 Gy	3228 Gy	2.81
**Planned Maintenance**	5574 Gy	1626 Gy	2619 Gy	2.91
**Daily Quality Assurance** (including daily monitor check for all energies)	1488 Gy	768 Gy	1075 Gy (1881 Gy)	1.09
**Other Quality Assurance**			2611 Gy	2.95

^a^ Average doses and ratios of dose‐to‐patient dose are calculated without the years 1998–2000 and 2006 when remodeling works in the operation theatre led to extremely small patient numbers.

### D.1 Daily safety checks and warm up

The dose consumption for daily safety checks and warm up is limited to three short measurements to confirm correct function of the:

- primary dose monitor
- dose timer
- manual rad off

The remaining tests requiring radiation (e.g. door interlocks, functionality of warning lights) are checked within the monitor tests, all others without beam. By these means the total radiation used for the safety check (under normal conditions) can be limited to 4 Gy per check. In the calculations, an additional dose of 4 Gy per check is added to check the calibration of the dose monitor for two energies: on every day a patient is scheduled, monitor constancy is checked for one standard energy (typically the most frequently used energy, 8 MeV) plus one additional, varying energy. Note that these are minimal doses, kept small with the intention to allow for a maximum number of patient treatments within a given (small) dose license for the OR. If additional daily actions under radiation (e.g. a daily check of monitor constancy for all energies) or a warm‐up dose are planned, the dose for daily checks can easily rise by a factor of 2–3, or more. Additionally, at times of instable beam, higher doses may be applied when repeated irradiations are necessary to confirm safety.

**Figure 3:**
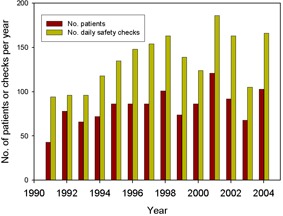
Numbers of daily safety checks performed in different years in comparison to the number of patient treatments.

Figure 3 shows the numbers of daily safety checks performed during the years 1991 to 2007 in comparison to the number patient treatments. The average number of patients per year is 75; the average number of checks per year is 134 (i.e. on average, 79% more checks are scheduled than patients treated).

### D.2 Biweekly check of monitor calibration constancy

**Figure 4:**
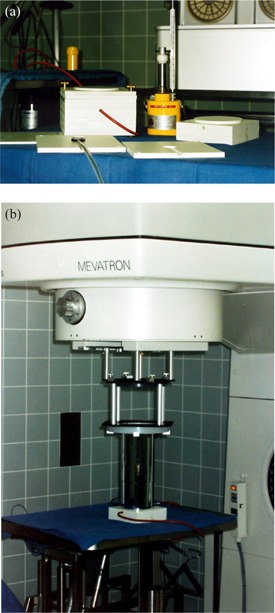
Dosimetry phantom (a) and setup (b) for the Mevatron ME.

Checks of dose monitor constancy for all energies and dose rates and, if necessary, re‐calibrations are performed biweekly using a 0.3 cm^3^ thimble ionization chamber in the phantom arrangement shown in Fig. 4. The white polystyrene phantom is shaped to fit into the end of the round (12 cm diam.) reference applicator and allows a quick and reproducible setup. Measuring depth is 2.5 cm. Note that for the small energies (6 and 8 MeV) also, energy shifts will be detected as dose deviations, so that energy constancy must be monitored in parallel. This is in part achieved by keeping record of accelerator parameters defining beam energy. These parameters are essentially the bending magnet current, the current of steering coils, and the size and shape of the monitor pulses.

A monitor check without recalibration consumes 6×12Gy=72Gy. Assuming an average of 25 checks per year, biweekly monitor calibrations will add 1800 Gy to annual dose consumption.

In cases where monitor adjustments are necessary, dose consumption rises by about 10–20 Gy for each energy adjusted. For the Mevatron ME, recalibration tends to be necessary 3–4 times per year. The recalibrations are not included in the summation in Table 3.

The phantom measurements are cross‐calibrated for all energies to a measurement of absorbed dose to water using a Marcus chamber in a water phantom. The cross‐calibration is regularly checked during the annual calibrations.

Biweekly checks of monitor calibration fulfill the requirements as described in the German DIN standards for quality assurance of medical electron accelerators used in radiotherapy,^(16)^ whereas TG‐40^(12)^ recommends a daily check of constancy and a monthly calibration. The need of only 3–5 recalibrations per year corresponds to an action threshold at dose deviations of ±3%. This low frequency is based on the high stability of the monitor systems in modern radiotherapy accelerators and gives reason for infrequent checks of monitor calibration at machines used in fractionated radiotherapy.

### D.3 Constancy of beam energy

**Figure 5:**
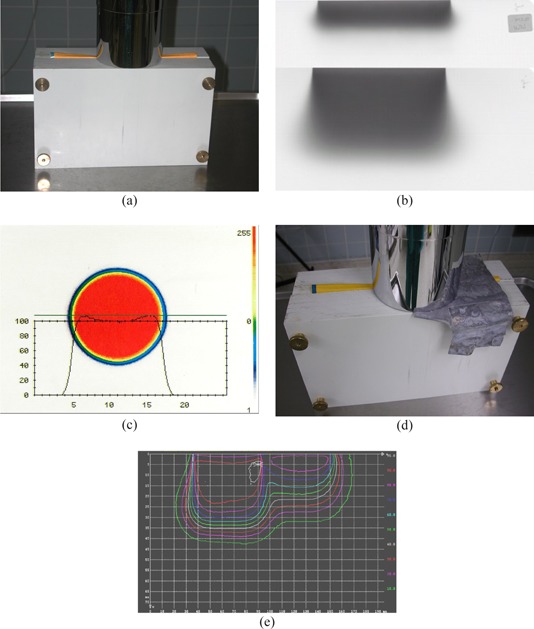
Phantom arrangement for film dosimetry at the Mevatron ME: (a) normal setup to simultaneously check depth dose, fatness, and symmetry; (b) dosimtery films exposed with 6 MeV (upper) and 18 MeV (lower) electrons; (c) dose profile and color‐wash dose distribution of a 12 cm round applicator measured with film dosimetry at depth of $D_{\max}$; (d) arrangement to measure the reduction of electron dose by 0.5 mm of lead; (e) dose distribution corresponding to the arrangement in (d).

Additional to keeping the energy‐defining accelerator parameters constant, checks of beam energy constancy are performed with film dosimetry approximately four times per year. The films (Kodak X‐omat V, in light‐tight envelopes) are cut in two and resealed with light‐tight black tape. The film halves fit into a white polystyrene phantom as shown in Fig. 5 that are positioned flush against the end of the reference cone. The part of the envelope overlapping the film is carefully folded aside, so that the film edge sits directly at the cone end without allowing any gaps. The film is positioned parallel to the beam axis through the center of the cone opening. With this arrangement, both a measure for the depth dose curve and field fatness and symmetry can be determined with one film. The depth extinction curve on film is compared to a reference curve which was determined immediately after a depth dose measurement using a Markus chamber in water. Parameters compared to the reference curve are the extinction equivalents of the electrons practical range $R_{p}$, depth of $D_{\max}$, surface dose, and bremsstrahlung background. Check criteria are constancy of the depth equivalents within ±2mm, and constancy of the dose equivalents within 3%–5%, depending on parameter.

Each film is irradiated with a dose of 0.8 Gy leading to a dose consumption per check of 6×0.8Gy=4.8Gy.

### D.4 Annual calibrations

Annual calibrations are performed for two cones, for which two OAR distributions and one PDD curve are measured for each cone and energy. To confirm beam stability at all gantry angles, the curves are measured at three gantry angles (0°, 90°, and 324°). Using the same assumptions as for commissioning, the dose consumption amounts to 270 Gy. Additionally, a monitor calibration in a water phantom is performed together with a cross‐calibration of the polystyrene phantom for biweekly monitor checks, using 216 Gy (including one repetition of each measurement). In total these measurements consume a dose of 486 Gy.

### D.5 Repairs and preventive maintenance

**Figure 6:**
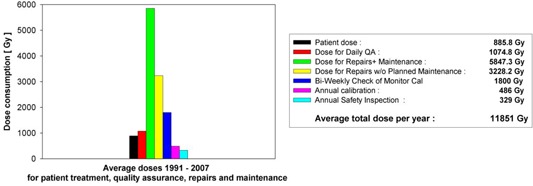
Average dose consumption for patient treatment, daily safety checks, repairs and maintenance, unplanned repairs and maintenance, annual calibrations, biweekly checks of monitor calibration, and annual safety inspections. Symbols for the bars are given in the box, which also shows the average doses for the different causes. The doses for daily safety checks contain 4 Gy per day for check of the monitor calibration.

Fig. 6 shows the doses used for patient treatments, daily safety checks, repairs, preventive maintenance, biweekly monitor calibration checks, and annual safety inspections, averaged over the 17 years of observation (1991 to 2007).

Patient doses were taken from the patient log (average 886 Gy per year). In two years where the exact number of patient numbers was not known, an average number of 75 patients was estimated and multiplied by an average dose of 12.5 Gy. The doses for daily safety checks contain 4 Gy for warm‐up and test of interlock systems plus an additional 4 Gy per day for checks of the monitor calibration. A daily calibration check for all six energies would increase the annual dose for this item from 1075 Gy to 1881 Gy. Doses for biweekly monitor calibration checks are always taken as 72Gy×25 checks. The other doses are calculated from the HV timer readings, as described above. For these calculations, an uncertainty of ±10%–20% is estimated. Uncertainties of ±10% come from rounding errors in the HV timer reading. A second source of uncertainty comes from always calculating the doses for a dose rate of 9 Gy/min and not correcting for the adjustments at lower dose rates. This part of the uncertainty will generally lead to an overestimation of dose in the range of 10%–20%.

Doses for all actions other than patient treatment are shown in Table 4. The Table shows the maximum and minimum doses consumed in one year of the observation period together with the average annual dose. Additionally the average ratio of doses to annual patient dose is given. The results are discussed in the following section.

**Figure 7:**
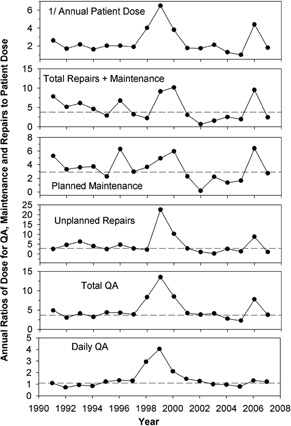
Annual ratios of dose for QA, maintenance and repairs to patient dose. The horizontal lines indicate the average ratios of the respective dose excluding the years 1998–2000 and 2006 which reported extremely small patient numbers.

To illustrate correlations between repairs and patient dose or time‐dependent trends in repairs, Fig. 7 shows the ratios of the annual doses for QA, maintenance, and repairs to the annual patient dose for each year of operation. For comparison, the plots also contain the respective average ratios. Fluctuations and trends in patient dose can be seen in the top plot in Fig. 7, which shows a curve of 1/ annual patient dose. For comparison to the lower plots, the curve is normalized to its minimum and the scale is adjusted to the lower scales. On average, this curve remains fairly constant over the years, so that any trend seen in other dose items may be attributed to the respective item. In 1998–2000 and in 2006, construction works in the operation theatre led to low patient numbers, and consequently low patient doses. The ratios therefore show peak values in these years. The peaks also appear in the other diagrams since repair and maintenance works were not reduced to the same extent. These years are not typical for normal operation and therefore not used in the calculation and the discussion of the average ratios. However, the high ratios in these years may be typical for some facilities with low patient numbers and high numbers of repairs.

### D.6 Leakage measurements

The results of the leakage radiation measurements are shown in Table 5.

**Table 5:** Leakage dose around the Mevatron ME.

*Absorption Depth in Phantom*	*90° Longitudinal 9 cm mSv*	*90° Lateral 9 cm mSv*	*0° 10 cm mSv*	*0° 15 cm mSv*	*30° From Beam 17.3 cm mSv*
Energy					
6 MeV	5.1	4.5	1.7	1.4	1.1
8 MeV	5.4	4.7	2.4	2.1	1.5
10 MeV	6.0	5.3	4.1	3.6	2.2
12 MeV	6.0	5.2	5.4	4.8	2.6
15 MeV	7.0	6.1	10.4	9.6	4.4

Doses are given in mSv per 10Gy beam dose at $d_{\max}$, using a factor of 1.3 to convert absorbed into equivalent dose. The measuring positions are shown in Fig. 1. The line absorption depth in phantom gives the thickness of absorbing phantom material measured from the reference point to the phantom surface in the direction of the measuring point.

## IV. DISCUSSION

The high doses connected with IORT produce leakage radiation on the order of several Sv per year (see Table 5). Radiation protection for a stationary accelerator located in an OR will probably require large amounts of structural shielding to protect the surrounding area, since one must expect there to be a variety of personnel and public presence which may be difficult to control. If an IORT program is to be introduced at a later time into an existing OR, high costs may emerge for add‐on shielding as well as long down times for remodeling the facility.

For modern mobile IORT accelerators, dose can be separated into patient‐related irradiations which must be performed in the OR, and others that can be performed somewhere else. With this strategy, the OR must provide radiation protection only for those irradiations which need to be performed in an uninterrupted sequence with patient treatment. Usually these will be: daily warm‐up, safety and monitor checks, and the treatment itself.

For all others irradiations, the machine can be moved to a different location, and the large amounts of dose necessary to maintain safe operation of the accelerator can be irradiated here. This second location may be an existing radiation vault in radiotherapy or a separate room which can be equipped with sufficient radiation shielding at lower costs than the OR. By these means, it may be possible to greatly reduce costs for structural shielding, down‐times for reconstruction works in a running OR, and many organizational impositions.

Irradiations which can take place in a separate vault are commissioning, QA other than daily checks, planned maintenance, repairs and development, and experimental works.

### A. Patient‐related irradiations which must be performed in the OR

Patient‐related doses depend mainly on the number of patients, the applied patient doses, and on machine properties which determine the doses needed for daily QA. At the Heidelberg Mevatron ME, daily QA (including a monitor check at two energies as describes above) leads to an average dose of 1075 Gy per year. As shown in Fig. 7 (bottom plot), this equals a fairly constant factor of 1.1 times patient dose in all years of normal operation. However, in the years with low patient numbers, the ratio rose to 4. The number of checks was on average 1.79 times the number of patients. This number decreases if more than one patient is treated per day of operation. At Heidelberg where no special provisions where taken for this cause, more than one patient was treated on 7% of treatment days. Under these conditions, smaller patient numbers will reduce daily QA proportionally. Possibly, more care in patient selection could reduce the ratio of scheduled to treated IORT patients.

QA doses also depend on machine characteristics such as the number of beam energies (or actually the number of independent monitor calibration circuits for the different energies, dose rates, etc.) and the time or dose necessary for the machine to produce a stable output. For daily QA of the Mevatron ME, a minimal dose of 8 Gy is needed for warm‐up, safety checks, and monitor checks at two energies. If one chooses to check all six energies, total dose per check would increase to 16Gy, assuming a dose of 2Gy per energy. Note that to perform monitor checks with doses much smaller than typical treatment dose, the accelerator should reach a stable output during short times, and monitor linearity must be sufficiently valid at small doses. Depending on monitor performance, with other accelerators different doses may be preferred. Therefore, publication of practical data for daily QA should be encouraged.

The experience at Heidelberg indicates that, without specific agreements to limit treatment days, twice the planned patient number checks should be a safe estimate of the number of daily QA.

Under these assumptions the dose in the OR can be estimated as:

$$\begin{array}{c} \text{Dose per week}=\text{No.}\;\text{of patients per week}\times (\text{average patient dose}+2\times \text{dose}\\ \;\text{for daily QA}). \end{array}$$

Daily QA dose can possibly be reduced if agreements are made to restrict IORT to a limited number of days per week or to treat more than one patient per day.

### B. Doses which can be irradiated in a separate vault

#### B.1 Commissioning

At Heidelberg, a dose of 5145 Gy was needed for these basic measurements. The largest part of this dose (4110 Gy) is needed for measurement of dose distributions, and cone and gap correction factors for all applicators at all energies. The data in Table 1 show that one can assume a dose of roughly 43 Gy per applicator and energy. The other items in Table 1 consumed a dose of 1035 Gy. Similar doses as found in this study should also apply to other accelerators, so that an estimate of commissioning dose may be calculated by:

  commissioning dose=(No.of applicators+No. of energies)×43−45Gy+1100Gy.

Note that commissioning happens during a short period. Therefore organizational measures may be necessary to accommodate high dose levels during a short time. This can possibly be achieved by temporal evacuation of surrounding areas.

If one chooses to perform commissioning in a multi‐use vault (e.g. the vault for another accelerator in radiotherapy), one should schedule the measurements so that they do not interfere with other works in the room.

### C. Pre‐plannable QA other than daily checks

For the QA measurements (without daily checks) at Heidelberg, a dose of 2630 Gy per year is needed for six energies. These doses are hardly reducible, since they are required by regulations, and will probably be similar for other types of electron accelerators.

#### C.1 Planned maintenance and unplanned repairs

At Heidelberg, these works consumed an average of 5847 Gy per year (maximum 9792 Gy, minimum 1197 Gy). As stated above, these doses may be overestimated by 10%–20%. No clear correlation of repair and maintenance doses to patient dose is visible in the curves in Fig. 7, except for the years with low patient numbers. A small decrease of dose with time for planned maintenance may be due to growing experience with the accelerator giving room for less extensive adjustments and controls. However, large parts of these doses are required either legally by safety regulations or by the maintenance policy of the accelerator manufacturer, so they can not readily be reduced. Since planned maintenance doses should be independent of patient numbers, one should estimate the dose for shielding calculations from the manufacturer's maintenance policy and existing legal requirements. For the Mevatron ME, planned maintenance consumed an average dose of 2500 Gy per year (min 1500 Gy, max 5500 Gy).

Doses for unplanned repairs are difficult to predict. The high doses for repairs found in this work may be reduced with the new designs of mobile IORT linacs. These machines are especially designed for electron‐only operation. A major difference to linacs running in X‐ray mode is that beam currents and RF loads are much smaller for electrons. On a dual mode machine, the electronics must therefore handle a wide range of signals. Wide band electronics tend to operate with less stability with the small electron signals. The new electron‐only machines may have electronics that are better adjusted to the small signals, and therefore the linac may need less beam adjustments and less repair time.

With respect to beam adjustments, the Mevatron ME proves to be especially problematic. The RF of the Mevatron ME is produced with a high‐power magnetron (EEV type MG5349, EEV Ltd., Waterhouse Lane Chelmsford Essex CM1 2QU England) which runs at very high currents in order to produce the high beam energies 15 and 18 MeV. The machine stress and instability from this mode of operation necessitates frequent beam adjustments and is, in part, the reason for a large number of repairs and a short lifetime of the magnetron.

In any case, high doses will occur for repair and maintenance for which the radiation protection of the dosimetry vault must be prepared. To estimate the dose, it is highly recommended that one obtain as much information as possible on the dose needed for repairs, maintenance, and QA before planning the radiation shielding of an IORT site. Publications of such data would be helpful.

Repair works will produce high doses during short times, so that the times to accumulate ambient dose limits must be assumed equally short. This may need to be considered when licensing permitted dose workloads per time. On the other hand, repair works can mostly be performed in the least critical position (e.g. with the beam directed straight down or in some other position which minimizes dose to critical areas).

Even for a mobile machine, a timely relocation of the accelerator into a separate shielded vault may conflict with busy OR schedules and department workload. Possibly the accelerator can not be moved from the OR for all repairs. Some work may technically require the machine to remain in the OR (e.g. for faults connected to installations within the OR); for others, time reasons may not allow moving the machine (e.g. due to a patient waiting for immediate treatment).

Irradiations for developments and experiments are planned works, and can usually be performed in the separate vault. Doses will vary for different institutions, and should be estimated individually

### D. Leakage measurements

The results of the leakage measurements agree with measurements by Mills et al.^(10)^ The measurements at 1 m horizontal from the reference point can be compared to Mill's measurements at 45° from beam axis (with origin at the scattering foil) and deliver comparable results (0.5% of the dose at the reference point as compared 0.7% found by Mills). At 0° Mills measures a higher leakage of 5% versus 0.1%. This may be due to the higher energy of 18 MeV measured by Mills (18 MeV was no longer in operation at time of measurements, to reduce stress on Magnetron). An additional reason may be that in order to measure at 1 m distance from the scattering foil, Mills has placed an acrylic absorber in the beam at a shorter distance, where the electron dose rate and consequently also leakage dose rate at the point of measurement is higher.

Compared to leakage doses published for the mobile accelerators (Mobetron, Liac), the doses measured for the Mevatron ME at 10 MeV in the horizontal plane are roughly a factor of 10 higher. This difference is mainly caused by reduced scatter in the accelerator head for the mobile machines, which are designed without a bending magnet for this purpose. In the forward direction, the differences are smaller (factor 3–6), probably due to larger leakage contributions from the collimation system, the applicator, and the phantom, which are present in all accelerators. Neutron leakage was not measured in this work. Neutron doses from IORT accelerators have been reported in several publications^(^ ^10^ ^,^ ^22^ ^–^ ^24^ ^)^ and contribute between $10^{-8}$ and $10^{-5}$ of reference dose.

## V. CONCLUSIONS

IORT is different from conventional external beam radiotherapy in that it is not mainly the number of patients and treatment dose that determine the dose for which the facility must be prepared. With an IORT accelerator, dose consumption for commissioning, repairs, maintenance, and QA is multiple of patient dose. The total dose consumption for all of these actions must be considered in facility licensing, in radiation protection assessments, and in the shielding calculations. For a stationary linac, the shielding of the OR must be dimensioned to reduce the leakage to the surrounding area from the complete annual dose, including QA and repairs, to levels permitted by local or national regulations.^(^ ^1^ ^,^ ^2^ ^)^ For the newly designed mobile IORT accelerators,^(^ ^4^ ^,^ ^5^ ^,^ ^6^ ^,^ ^7^ ^)^ costs and time can be saved by performing those parts of the dose which can be scheduled in advance in a shielded vault separate from the OR. Also this vault must be licensed and its shielding must be assessed in advance. In contrast to estimated patient dose, the dose consumption for QA, repairs, and maintenance is usually not readily known in the planning phase. For this purpose, additional to published recommendations on QA, publications are also needed which report on practical experience and dose in QA, maintenance, repairs, and leakage for every type of IORT device.

## Footnotes

1 To reduce stray radiation to an adjacent hallway, beams in this direction are limited to gantry angles larger than 324°.

2 The dose difference of 544 Gy corresponds to a measuring time of 3.02 hours at 3 Gy/min. Since the commissioning program included the measurement of 1314 PPD and OAR curves, an over‐estimation of measuring time of 8 seconds per curve would result in the observed dose / timer difference.
